# Measuring the Burden of Neglected Tropical Diseases: The Global Burden of Disease Framework

**DOI:** 10.1371/journal.pntd.0000114

**Published:** 2007-11-07

**Authors:** Colin D. Mathers, Majid Ezzati, Alan D. Lopez

**Affiliations:** 1 Information, Evidence and Research Cluster, World Health Organization, Geneva, Switzerland; 2 Harvard School of Public Health and Harvard University Initiative for Global Health, Cambridge, Massachusetts, United States of America; 3 School of Population Health, University of Queensland, Brisbane, Queensland, Australia; London School of Hygiene & Tropical Medicine, United Kingdom

## Abstract

Reliable, comparable information about the main causes of disease and injury in populations, and how these are changing, is a critical input for debates about priorities in the health sector. Traditional sources of information about the descriptive epidemiology of diseases, injuries, and risk factors are generally incomplete, fragmented, and of uncertain reliability and comparability. The Global Burden of Disease (GBD) study has provided a conceptual and methodological framework to quantify and compare the health of populations using a summary measure of both mortality and disability, the disability-adjusted life year (DALY).

This paper describes key features of the Global Burden of Disease analytic approach, which provides a standardized measurement framework to permit comparisons across diseases and injuries, as well as risk factors, and a systematic approach to the evaluation of data. The paper describes the evolution of the GBD, starting from the first study for the year 1990, summarizes the methodological improvements incorporated into GBD revisions for the years 2000–2004 carried out by the World Health Organization, and examines priorities and issues for the next major GBD study, funded by the Bill & Melinda Gates Foundation, and commencing in 2007.

The paper presents an overview of summary results from the Global Burden of Disease study 2002, with a particular focus on the neglected tropical diseases, and also an overview of the comparative risk assessment for 26 global risk factors. Taken together, trypanosomiasis, Chagas disease, schistosomiasis, leishmaniasis, lymphatic filariasis, onchocerciasis, intestinal nematode infections, Japanese encephalitis, dengue, and leprosy accounted for an estimated 177,000 deaths worldwide in 2002, mostly in sub-Saharan Africa, and about 20 million DALYs, or 1.3% of the global burden of disease and injuries. Further research is currently underway to revise and update these estimates.

## Introduction

Governments and international agencies are faced with setting priorities for health research and investment in health systems and health interventions in a context of increasing health care costs, increasing availability of effective interventions, and numerous and diverse priorities and interest groups. Evidence on the magnitude and trends of diseases and their causes should be a critical input to decision making at the global, national, and local levels. Broad evaluation of the effectiveness of health systems and major health programs and policies also requires assessments of the causes of loss of health that are comparable not only across populations, but also over time.

The World Bank's 1993 World Development Report on *Investing in Health* recommended cost-effective intervention packages for countries at different levels of development [1]. Underpinning these analyses was the first Global Burden of Disease (GBD) study, carried out by Chris Murray at Harvard University and Alan Lopez at the World Health Organization (WHO), in collaboration with a global network of over 100 scientists [1]–[4]. To produce comprehensive, valid, reliable, and comparable information of maximum relevance to decision making, the GBD analytic framework included several novel attributes:

- Information on causes both of premature mortality and of morbidity, impairment, and disability was combined to present a balanced, comprehensive assessment of health problems. This helps appropriately represent the impact of conditions such as onchocerciasis, trachoma, filariasis, intestinal helminthes, schizophrenia, depression, and paralysis, which cause great suffering and loss of health but little mortality.
- The study used a standard unit of health measurement, namely disability-adjusted life years (DALYs). The results can then easily be incorporated into comparisons of costs and effects of different interventions to reduce the burden of disease. Use of a common metric also facilitates the quantification of disease burden from both diagnostic categories of the International Classification of Disease and Injuries (ICD), and the major risk factors that cause those health outcomes.
- All disease and injury causes were included in the analysis (this avoids the problem of over-inclusiveness of single cause studies, and of incompatible mortality claims for different causes). This in turn required the development of methods to estimate missing data.

This paper describes the GBD framework for integrating, validating, analyzing, and disseminating fragmentary information on the health of populations so that it is truly useful for health policy and planning.

## Global Burden of Disease 1990 Study

The original GBD study was commissioned in 1992 by the World Bank to provide a comprehensive assessment of disease burden in 1990 from more than 100 diseases and injuries, and from 10 selected risk factors for the world and eight major World Bank regions [1]–[3], [5]–[7]. Earlier attempts to quantify global cause of death patterns [8],[9] had been largely restricted to broad cause of death groups.

As well as generating consistent estimates of mortality, incidence, prevalence, and disability for over 130 causes by age, sex, and world region, the GBD study introduced a new metric—the DALY—which summarized the loss of health due to mortality and morbidity combined. The DALY is examined in more detail in the following section. Much of the comment and criticism of the GBD study has focused on the construction of DALYs [10]–[13], particularly the social choices around age-weights and severity scores for disabilities, and relatively little around the large uncertainty in the basic descriptive epidemiology, especially in Africa, which is likely to be far more consequential for setting health priorities [14]. These criticisms are examined in more detail in the Discussion section below.

The GBD study developed methods for assessing causes of burden for which there were limited data and considerable uncertainty, to ensure that such causes were not implicitly considered to have zero burden. To prepare estimates of the incidence, prevalence, duration, and mortality from over 500 sequelae of more than 100 diseases or injuries, a mathematical model, DISMOD, was developed for the 1990 GBD study to convert partial, often nonspecific data on disease/injury occurrence into a consistent age description of the basic epidemiological parameters in each region [15].

The leading causes of disease burden in 1990 were childhood diseases (lower respiratory diseases, diarrhoeal diseases, and perinatal causes such as birth asphyxia, birth traumas, and low birth weight), in part because of the greater weight given to deaths at younger ages by the DALY. Depression ranked fourth globally, ahead of ischemic heart disease, cerebrovascular disease, tuberculosis, and measles. Road traffic injuries also ranked in the top 10 causes of DALYs worldwide. The results of the original GBD study were surprising to many health policy makers, who were more familiar with the pattern of causes represented in mortality statistics. Neuropsychiatric disorders and injuries were major causes of lost years of healthy life as measured by DALYs, and were greatly undervalued when measured by mortality alone [4],[XPATH ERROR: unknown variable "next".]. More broadly, noncommunicable diseases, including neuropsychiatric disorders, were estimated to have caused 41% of the global burden of disease in 1990, only slightly less than communicable, maternal, perinatal, and nutritional conditions combined (44%), with 15% due to injuries.

The methods and findings of the original (1990) GBD study have been widely published [1]–[5],[16], and the GBD approach has been widely adopted by countries and health development agencies alike as the standard for health accounting. The methods and findings of the original GBD study stimulated quite a number of national disease burden studies of varying scope and methodological rigour during the 1990s. The earliest comprehensive studies were undertaken for Mexico and Mauritius [17],[18], followed by studies in the late 1990s in the Netherlands and Australia [19]–[22]. In the last few years, comprehensive national burden of disease studies have also been carried out in countries such as Brazil, Malaysia, Turkey, South Africa, Zimbabwe, Thailand, and the United States, and studies are underway in Canada and several other countries.

## The DALY—Construction and Concepts

To assess disease burden, a time-based metric that measured both premature mortality (years of life lost, or YLLs) and disability (years of life lived with a disability, weighted by the severity of the disability, or YLDs) was developed for the GBD 1990 study [23]. The sum of the two components, namely DALYs, provides a measure of the future stream of healthy life lost as a result of the incidence of specific diseases and injuries in 1990. One lost DALY can be thought of as one lost year of “healthy” life (either through death or illness/disability), and total DALYs (the burden of disease) as a measurement of the gap between the current health of a population and an ideal situation where everyone in the population lives into old age in full health. A more complete account of the DALY, and the value choices it incorporates, is given elsewhere [23]–[25]. DALYs are a particular formulation of the more general quality-adjusted life years (QALYs) measure proposed by Zeckhauser and Shepard in 1976 and widely used in cost-effectiveness analyses for health interventions [26]. DALYs measure health loss in populations against a normative standard, whereas QALYs are usually used to quantify health gains for interventions. For cost-effectiveness analyses, the mechanics of estimating DALYs averted and QALYs gained are virtually identical [27]. The approaches potentially differ only in the quantification and interpretation of the weighting system (discussed further in the Discussion below).

**Figure 1:**
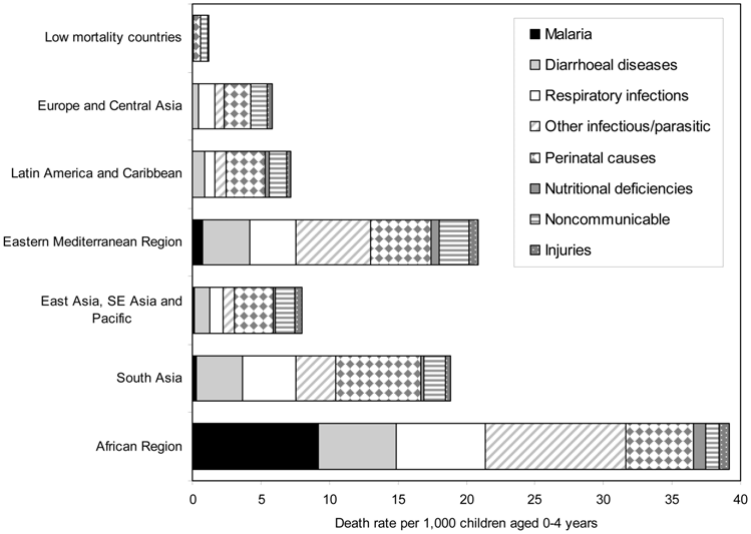
Death rates by broad cause group and region, children aged 0–4, 2002.

The YLLs for deaths at a given age *x* are calculated from the number of deaths, *d_x_*, at that age multiplied by a global standard life expectancy, *L_x_*, which is a function of age *x.* The GBD 1990 study chose not to use an arbitrary age cut-off such as 65 or 70 years in the calculation of YLLs, but rather specified the loss function *L_x_* in terms of the life expectancies at various ages in standard life tables, with life expectancy at birth fixed at 82.5 years for females and 80.0 years for males (Figure 1). The loss function was specified to be the same for all deaths of a given age and sex, in all regions of the world. This standard has continued to be used, and should not be confused with the country-specific life tables estimated for all WHO Member States for 2002, which summarize all-cause mortality rates in 2002 by age and sex.

YLDs for a particular cause in a particular time period are calculated by multiplying the number of incident cases *i_x_*, at each age *x* in that period, by the average duration of the disease for each age of incidence, *l_x_*, and a weight factor d*w_x_*, that reflects the severity of the disease on a scale from 0 (full health) to 1 (dead). YLDs are generally calculated either for the average incident case of the disease, or for one or more disabling sequelae of the disease. For example, YLDs for onchocerciasis are calculated by adding the YLDs for the sequelae of low vision, blindness, and itchy dermatitis. The “valuation” of time lived in nonfatal health states formalizes and quantifies social preferences for different states of health as *disability weights (dw_x_)*. Disability weights are further discussed later in this paper.

Murray and Lopez chose to apply a 3% time discount rate to the years of life lost in the future to estimate the net present value of years of life lost in calculating DALYs, and also incorporated nonuniform age weights that gave less weight to years of healthy life lost in early childhood or at older ages [23]. Time discounting is applied to the years of life lost in the future for consistency with the measurement of health outcomes in cost-effectiveness analyses; to prevent giving excessive weight to deaths at younger ages; and to address the disease eradication and research paradox. Assuming that investment in research or disease eradication has a nonzero chance of succeeding, then without discounting, all current health expenditure should be shifted to such investment because the future stream of benefits is infinite [28].

The introduction of nonuniform age weights was based on human capital arguments and on a number of studies that suggest the existence of a broad social preference to value a year lived by a young adult more highly than a year lived by a young child or an older person. The particular age weights used in the GBD study result in greater weight being given to all deaths below age 39 compared with deaths at older ages. When discounting and age weighting are both applied, a death in infancy corresponds to 33 DALYs, while deaths at ages five to 20 equate to around 36 DALYs [28].

## GBD 2000–2004: Improved Methods, More Data

From 1999 to 2004, WHO published an annual update of the GBD in the *World Health Report* Annex tables (see for example [29]). The update of the GBD for the year 2000 was a major input to the assessment of healthy life expectancy for WHO Member States, used as one of the outcome measures to quantify health system performance in 2000 [30]. A major expansion of the work on risk factors was released in the *World Health Report 2002* [31] and in subsequent detailed volumes in 2004 [32]. The GBD results for the year 2001 provided a framework for cost-effectiveness and priority setting analyses carried out for the Disease Control Priorities Project (DCPP), a joint project of the World Bank, WHO, and the National Institutes of Health, funded by the Bill & Melinda Gates Foundation [33]. The GBD results were documented in detail, with information on data sources and methods as well as uncertainty and sensitivity analyses, in a book published as part of the DCPP [25].

While the first GBD study was a major milestone for measuring population health at the global level, its value for comparative analysis was seriously limited by its use of only eight regions as the basic units of analysis. A more refined approach was followed for the GBD 2000 study. Country-level life tables and mortality estimates by disease and injury cause, age, and sex were first developed for each of the 192 WHO Member States using different methods for countries with different availability of mortality data. Incidence, prevalence, and YLD estimates were developed for 17 epidemiological groupings of countries, and then imputed to country populations using available country-level information and methods to ensure consistency with the country-specific mortality estimates. The resulting country-level estimates were made available by WHO at a summarized level, and also facilitated the production of regional estimates for any required groupings of countries. The production of country-level estimates also enabled substantially more engagement with countries as a starting point for health situation assessments and national burden of disease analyses.

New methods were developed for a number of components of the GBD 2000, including: a new system of model life tables for estimating age-specific death rates [34], better methods for modeling the relationship between the level of mortality and the broad cause structure in populations without complete death registration data [35], better and more consistent methods for calculating mortality and burden of disease attributable to major risk factors, individually and in combination [36], and more systematic approaches to the assessment of uncertainty [28]. Even more importantly, there was a substantial increase in primary data collected in developing countries, improved population surveillance for some major diseases such as HIV/AIDS, and wider availability of data from “verbal autopsy” methods, particularly in Africa, India, and China [37].

Death registration data were available for 107 countries, the majority of these in the high-income group, Latin America and the Caribbean, and Europe and Central Asia. Population-based epidemiological studies, disease registers, and notifications systems (in excess of 2,700 datasets) also contributed to the estimation of mortality due to 21 specific communicable causes of death, including HIV/AIDS, malaria, tuberculosis, childhood immunizable diseases, schistosomiasis, trypanosomiasis, and Chagas disease. Almost one-third of these datasets related to sub-Saharan Africa.

Estimating YLDs requires systematic assessments of the available evidence on incidence, prevalence, duration, and severity of a wide range of conditions, often based on inconsistent, fragmented, and partial data available from different studies. For each disease and injury included in the GBD, a limited set of disabling sequelae was selected to be evaluated in depth. Table 1 lists the disabling sequelae associated with malaria and specific neglected tropical diseases for which YLD estimates were prepared in the GBD 2000. Clearly, there are other sequelae for some of these conditions that have not been directly evaluated. Additionally, due to the limitations of cause assignment, particularly for deaths, cancers and some other chronic disease long-term sequelae are not redistributed to the initiating infectious disease in the primary GBD cause tabulations (such re-attributions need to be done using the counterfactual methods discussed later in this paper). The sequelae listed in Table 1 were selected in consultation with collaborating disease experts for direct evaluation and represent an attempt to include all important disabling outcomes while taking into account the limitations in the available data and evidence.

**Table 1:** GBD Cause Categories, Disabling Sequelae, and Average Disability Weights, for Malaria and Neglected Tropical Diseases

GBD Cause/Sequelae	Case Definition	Disability Weight	
		Average	Range
Malaria	Infectious disease caused by protozoa of the genus *Plasmodium*	0.191	0.172–0.211
Episodes	Attacks of chills, fever, and sweating due to *Plasmodium* infection	0.471	0.443–0.471
Anemia	Defined using WHO criteria for mild to very severe anemia	0.012	0.012–0.013
Neurological sequelae	Includes hemiplegia, aphasia, ataxia, and cortical blindness	0.350	
Trypanosomiasis—Episodes	Infection with protozoa of the genus *Trypanosoma,* excluding *Trypanosoma cruzi*	0.191	0.172–0.211
Chagas disease	Infection with *T. cruzi*		
Infection	Episode of infection with *T. cruzi*	0.000	
Cardiomyopathy without congestive heart failure	Disorder of the heart muscle resulting from infection with *T. cruzi* without congestive heart failure	0.062	
Cardiomyopathy with congestive heart failure	Disorder of the heart muscle resulting from infection with *T. cruzi* with congestive heart failure	0.270	0.186–0.308
Megaviscera	Dilation of interior organ in the abdominal cavity, particularly of esophagus and colon due to *T. cruzi*	0.240	
Schistosomiasis—Infection	Infection and associated direct mortality from schistosomiasis; does not include estimates of mortality from bladder cancer, cirrhosis, or colon cancer that may be related to schistosomiasis	0.006	0.005–0.006
Leishmaniasis	Infection with flagellate protozoa of the genus *Leishmania*		
Visceral	Generalized involvement of the reticuloendothelial system	0.243	
Cutaneous	Presence of skin lesions (which may ulcerate)	0.023	
Lymphatic filariasis	Infection with filariae (*Wucheria bancrofti* and *Brugia malayi*)		
Hydrocele > 15 cm	Circumscribed collection of fluid in testicle or along the spermatic cord	0.073	0.066–0.075
Bancroftian lymphedema	Swelling of subcutaneous tissues due to the presence of excessive lymph fluid as a result of infection with *Wucheria bancrofti*	0.106	0.067–0.128
Brugian lymphedema	Swelling of subcutaneous tissues due to the presence of excessive lymph fluid as a result of infection with *Brugia malayi*	0.116	0.064–0.128
Onchocerciasis	Infection with worms of the genus *Onchocerca*		
Blindness	Inability to distinguish the fingers of a hand at the distance of 3 meters, or less than 5% of remaining vision as compared to a normally sighted individual as a result of infection with *Onchocerca volvulus*	0.600	
Itching	Itchy dermatitis as a result of infection with *Onchocerca volvulus*	0.068	
Low vision	Corrected visual acuity in the better eye of less than 6/18 but better than or equal to 3/60 due to infection with *Onchocerca volvulus*	0.260	
Leprosy	Chronic disease resulting from infection with *Mycobacterium leprae*		
Cases	Person showing clinical signs of leprosy, with or without bacteriological confirmation of the diagnosis, and requiring chemotherapy	0.000	
Disabling leprosy	Grade 1 and 2 of WHO grades of disability for leprosy	0.152	
Dengue	Mosquito-borne disease caused by viruses of the family *Flaviviridae*		
Dengue hemorrhagic fever	Severe manifestation of dengue infection characterized by multiple hemorrhages, and potentially followed by circulatory failure, neurological manifestations, and shock	0.210	0.195–0.211
Japanese encephalitis (JE)	Mosquito-borne encephalitis caused by JE virus		
Episodes	Episode of JE infection	0.616	0.613–0.616
Cognitive impairment	Reduced cognitive function resulting from encephalitis due to JE virus	0.468	0.402–0.484
Neurological sequelae	Neurological deficits resulting from encephalitis due to JE virus	0.380	0.339–0.460
Trachoma	Cases of follicular or inflammatory trachoma		
Blindness	Corrected visual acuity in the better eye of less than 3/60	0.600	
Low vision	Corrected visual acuity in the better eye of less than 6/18 but better than or equal to 3/60	0.278	0.227–0.282
Ascariasis	Infection with worms of the genus *Ascaris*		
High-intensity infection	Infection resulting in at least 20–40 worms per stool load	0.000	
Contemporaneous cognitive deficit	Reduction in cognitive ability in school-age children, which occurs only while infection persists	0.006	
Cognitive impairment	Delayed psychomotor development and impaired performance on language skills, motor skills, and coordination equivalent to a 5–10 point deficit in IQ.	0.463	
Intestinal obstruction	Blockage of the intestines due to worm mass	0.024	
Trichuriasis	Infection with worms of the genus *Trichuris*		
High-intensity infection	Infection resulting in at least 250–500 worms per stool load	0.000	
Contemporaneous cognitive deficit	Reduction in cognitive ability in school-age children, which occurs only while infection persists	0.006	
Massive dysentery syndrome	Rectal prolapse and/or tenesmus and/or bloody mucoid stools due to carpeting of intestinal mucosa by worms	0.116	0.114–0.138
Cognitive impairment	Delayed psychomotor development and impaired performance on language skills, motor skills, and coordination equivalent to a 5-10 point deficit in IQ.	0.024	
Hookworm disease	Ancylostomiasis and necatoriasis		
High-intensity infection	Infection resulting in at least 80–160 worms per stool load	0.000	
Anemia	Anemia due to hookworm infection. Moderate or greater levels of anaemia are defined as haemoglobin of<100 g/l in pregnant women, <110 g/l in children and adult women and <120 g/l in adult men.	0.024	
Cognitive impairment	Delayed psychomotor development and impaired performance on language skills, motor skills, and coordination equivalent to a 5-10 point deficit in IQ	0.024	

Source: Mathers et al. [37], Annex Tables 3A.5 and 3A.6.

Data sources for the GBD 2000 study included WHO disease databases, national disease registers, epidemiological studies, health surveys, and health facility data. Around 8,700 datasets were used to quantify the YLD estimates for GBD 2000, of which more than 7,000 related to communicable, maternal, perinatal, and nutritional conditions. One-quarter of the datasets relate to populations in sub-Saharan Africa, and around one-fifth to populations in high-income countries. Details of data sources and methods for specific causes are available elsewhere [37].

While the GBD 2000–2004 drew on substantially more data for both mortality and epidemiological estimates, new systematic reviews and estimates were not completed for all causes, and some, such as dengue and Japanese encephalitis, continued to rely on the original GBD assessments of the mid-1990s. Additionally, YLD estimates for most causes continued to be based on the disability weights estimated for the original GBD study [23]. These weights were estimated using two forms of the person trade-off method and asked participants in weighting exercises to make a composite judgment about the severity distribution of the condition and the relative value of (or preference for) each severity level on a scale where 0 represents full health and 1 a health state equivalent to death the preference for time spent in each severity level. This was largely necessitated by the lack of population information on the severity distribution of most conditions at the global and regional levels. The participants were not representative of general populations, but were by and large public health professionals involved in a WHO meeting with representation from all regions and in training workshops held in several different regions. Issues in estimation of disability weights are further discussed in the Discussion section below.

## Disease and Injury Burden in 2002: An Overview

We present a brief overview of the results of the GBD Study for 2002 here. The findings for other years over the period 2000–2004 are very similar. Country groups used in the presentation of these results are shown in Supplementary Table S1. Slightly over 57 million people died in 2002, 10.4 million (or nearly 20%) of whom were children younger than five years of age. The risk of a child dying before age five ranged from 17% in sub-Saharan Africa to 0.7% in high-income countries (Figure 1). Low- and middle-income countries accounted for 99% of global deaths among children under the age of five years, and 85% of these were in the low-income countries. Only five preventable conditions—pneumonia, diarrheal diseases, malaria, measles, and causes arising in the perinatal period (primarily prematurity, birth asphyxia and trauma, and severe neonatal infections) were responsible for 70% of all child deaths.

**Figure 2:**
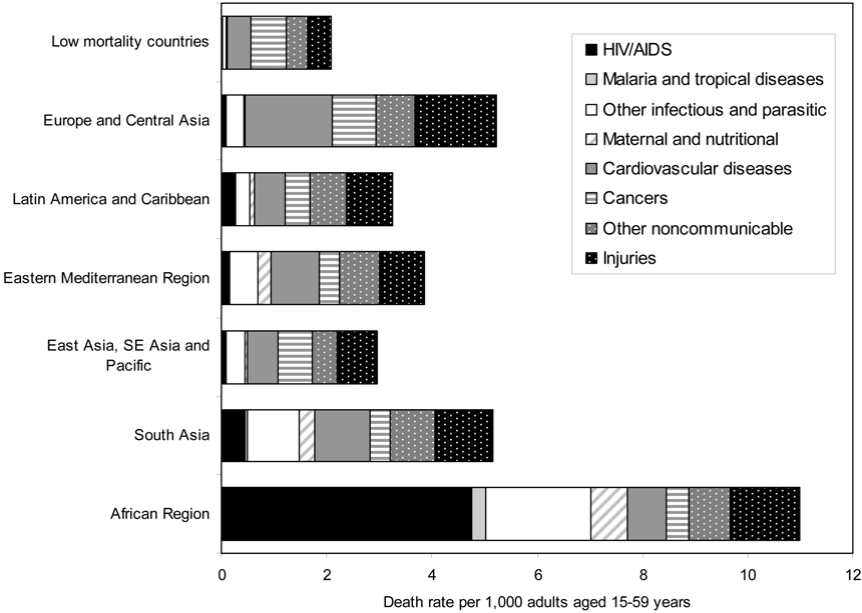
Death rates by broad cause group and region, adults aged 15–59, 2002.

In developing countries, noncommunicable diseases were responsible for more than 50% of deaths in adults aged 15–59 in all regions except South Asia and the African region, where communicable, maternal, perinatal, and nutritional conditions remained responsible for one-third and two-thirds of deaths, respectively (Figure 2).

Table 2 summarizes the 20 leading causes of death and of burden of disease globally in 2002. Ischemic heart disease and cerebrovascular disease (stroke) were the leading causes of death in both high-income countries and low- and middle-income countries in 2002, together responsible for more than 20% of all deaths worldwide. Four of the top 10 causes of death in the world were related to smoking (ischemic heart disease, stroke, chronic obstructive pulmonary disease, and lung cancer). In developing countries, five of the leading 10 causes of death were infectious diseases, including lower respiratory infections, HIV/AIDS, diarrheal diseases, tuberculosis, and malaria.

**Table 2:** The 20 Leading Causes of Deaths and Burden of Disease for the World, 2002

Leading Causes of Death				Leading Causes of Burden of Disease				
Rank	Cause	Deaths (millions)	Percent of total deaths	Rank	Cause		DALYs (millions)	Percent of total DALYs
1	Ischemic heart disease	7.21	12.6%	1	Perinatal conditions	97		6.5%
2	Cerebrovascular disease	5.51	9.7%	2	Lower respiratory infections	91		6.1%
3	Lower respiratory infections	3.88	6.8%	3	HIV/AIDS	84		5.7%
4	HIV/AIDS	2.78	4.9%	4	Unipolar depressive disorders	67		4.5%
5	Chronic obstructive pulmonary disease	2.75	4.8%	5	Diarrheal diseases	62		4.2%
6	Perinatal conditions	2.46	4.3%	6	Ischemic heart disease	59		3.9%
7	Diarrheal diseases	1.80	3.2%	7	Cerebrovascular disease	49		3.3%
8	Tuberculosis	1.57	2.7%	8	Malaria	46		3.1%
9	Malaria	1.27	2.2%	9	Road traffic injuries	39		2.6%
10	Trachea, bronchus, lung cancers	1.24	2.2%	10	Tuberculosis	35		2.3%
11	Road traffic injuries	1.19	2.1%	11	Chronic obstructive pulmonary disease	28		1.9%
12	Diabetes mellitus	0.99	1.7%	12	Congenital anomalies	27		1.8%
13	Hypertensive heart disease	0.91	1.6%	13	Hearing loss, adult onset	26		1.7%
14	Self-inflicted injuries	0.87	1.5%	14	Cataracts	25		1.7%
15	Stomach cancer	0.85	1.5%	15	Measles	21		1.4%
16	Cirrhosis of the liver	0.79	1.4%	16	Violence	21		1.4%
17	Nephritis and nephrosis	0.68	1.2%	17	Self-inflicted injuries	21		1.4%
18	Colon and rectum cancers	0.62	1.1%	18	Alcohol use disorders	20		1.4%
19	Liver cancer	0.62	1.1%	19	Protein-energy malnutrition	17		1.1%
20	Measles	0.61	1.1%	20	Falls	16		1.1%

Source: World Health Organization [29]

**Figure 3:**
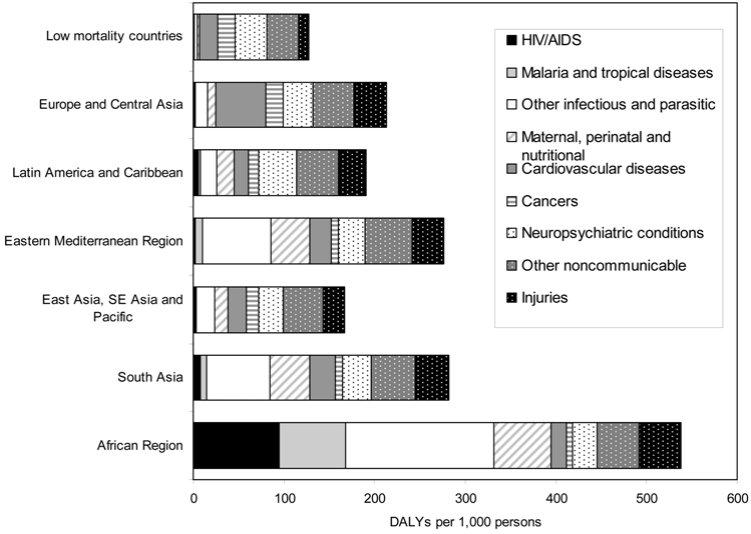
The burden of disease, by broad cause group and region, 2002.

HIV/AIDS has become the third leading cause of burden of disease globally, and the leading cause in sub-Saharan Africa, followed by malaria (Table 2). Communicable, maternal, perinatal, and nutritional conditions accounted for 73% of the burden of disease in sub-Saharan Africa, and 47% in South Asia (Figure 3). In other low- and middle-income regions, communicable, maternal, perinatal, and nutritional conditions accounted for a little under one-quarter of the disease burden. Total disease burden in Europe and Central Asian countries increased by nearly 40% over the period since 1990 and was higher in 2002 than for other developing regions of the world, apart from South Asia and sub-Saharan Africa. This increase reflects the sharp rise in adult male mortality and disability in the 1990s, related to cardiovascular disease and alcohol abuse in particular [38].

The overall burden of nonfatal disabling conditions is dominated by a relatively small set of causes. In all regions, neuropsychiatric conditions were the most important causes of disability, accounting for over 37% of YLDs among adults aged 15 years and over. Vision disorders, hearing loss, and musculoskeletal disorders were also important causes of YLDs, in both developed and developing countries.

Malaria, and the neglected tropical diseases specifically estimated as separate causes in the GBD (listed in Table 3), accounted for 1.3% of the global burden of disease (measured in DALYs) and 2.7% of global YLDs. The neglected tropical diseases listed in Table 3, excluding malaria, accounted for 18% of YLDs in the African region, with lymphatic filariasis contributing most from this group. In comparison, malaria accounted for 4.5% of YLDs in Africa. Table 3 also summarizes GBD estimates of global incidence and prevalence for malaria and 13 tropical diseases, many of which might be considered “neglected.” Also shown are estimates of global deaths, YLLs, YLDs, and DALYs for these diseases.

**Table 3:** Tropical Disease Mortality and Burden, Priority and Neglected Diseases, World, 2002

Disease	Incidence (000s)	Prevalence (000s)	Deaths (000s)	YLLs (000s)	YLDs (000s)	DALYs (000s)	YLLs per Death	YLDs per Case
Malaria—acute episodes	408,250	4,406	1,272	41,507	4,979	46,486	33	0.01
Lymphatic filariasis			0	10	5,768	5,777	23	3.69
Hydrocele>15 cm	1,564	38,137						
Bancroftian lymphedema	798	18,953						
Brugian lymphedema	150	3,434						
Trachoma			0	3	2,326	2,329	18	5.32
Blindness	437	2,936						
Low vision	400	3,517						
Leishmaniasis			51	1,569	521	2,090	31	0.98
Visceral	534	1,508						
Cutaneous	1,157	2,157		-	-	-		
Ascariasis—high-intensity infection	-	58,147	3	121	1,696	1,817	36	0.03
Schistosomiasis—infection	5,733	248,248	15	171	1,531	1,702	11	0.27
Trypanosomiasis—episodes	46	200	48	1,429	96	1,525	30	2.11
Trichuriasis—high-intensity infection	-	26,624	3	106	900	1,006	35	0.03
Hookworm—high-intensity infection	-	59,999	3	51	922	973	17	0.02
Japanese encephalitis—episodes	44	24	14	406	303	709	29	6.94
Chagas disease—infection	217	10,137	14	185	481	667	13	2.22
Dengue hemorrhagic fever	73	75	19	609	6	616	33	0.09
Onchocerciasis			0	0	484	484	22	8.72
Blindness	38	349						
Itching	56	1,346						
Low vision	47	601						
Leprosy—cases	175	903	6	86	113	198	14	0.65

Source: World Health Organization [29]

Mathers et al. [28] have made a partial and semi-quantitative assessment of uncertainty ranges for GBD estimates. The uncertainty range for malaria mortality and YLLs was estimated at around 30%, and uncertainty ranges for neglected tropical diseases with lower case fatality rates are likely to be even larger. Uncertainty in YLD estimates is due to both uncertainty in average disability weights (important particularly for high-prevalence sequelae with low average weights) and to uncertainty in incidence and prevalence estimates (important particularly for focal diseases where there is considerable uncertainty about populations at risk). Mathers et al. (Table 5.6) concluded that uncertainty in disability weights was particularly important for schistosomiasis, leishmaniasis, lymphatic filariasis, dengue, and the intestinal nematode infections [28]. Although the estimates in Table 3 have large uncertainty ranges, they do provide useful information on broad relativities of disease burden, on the relative importance of mortality and disability, and on regional patterns of disease burden.

Road traffic injuries were among the top 10 causes of DALYs for both high-income and low- and middle-income countries. Violence was also the fourth leading cause of burden in Latin America and Caribbean countries. In these countries, as well as in the Europe and Central Asian region, and the Middle East and North Africa, more than 30% of the entire disease and injury burden among male adults aged 15–44 was attributable to injuries, including road traffic injuries, violence, and self-inflicted injuries. Additionally, injury deaths were noticeably higher for women in some parts of Asia and the Middle East and North Africa, in part due to high levels of suicide and violence.

The GBD results clearly illustrate the “double burden” of disease faced by the poorer developing countries of South Asia and Africa. Countries that are still struggling with “old” and “new” infectious disease epidemics must now also deal with the emerging epidemics of noncommunicable disease such as heart disease, stroke, diabetes, and cancer.

## Comparative Quantification of the Burden of Disease from Risk Factors

Perhaps the most important methodological progress since the GBD 1990study has been made with respect to quantification of disease burden caused by risk factors. In the initial study, the population health effects of 10 risk factors were quantified, but there was limited emphasis on the comparability of the estimates. Different risk factors have very different epidemiological traditions, particularly with regard to defining “hazardous” exposure, the strength of evidence on causality, and the availability of epidemiological research on exposure and outcomes. Moreover, classical risk factor research has treated exposures as dichotomous, labeling individuals as either exposed or nonexposed, with exposure defined according to some, often arbitrary, threshold value. Recent evidence for such continuous exposures as cholesterol, blood pressure, and body mass index (BMI) suggests that such arbitrarily defined thresholds are inappropriate, since hazard functions for these risks rise (or decline) continuously across the entire range of measured exposure levels, with no obvious threshold [39].

For the GBD 2000 study, a new framework for quantifying risk factor burden was defined that measured changes in disease burden that would be expected under different population distributions of exposure [40]. Fractions of disease burden attributable to a risk factor were then calculated based on a comparison of disease burden expected under the current (i.e., 2000) estimated distribution of exposure, by age, sex, and region, with disease burden expected if a *counterfactual* distribution of exposure had applied. To improve comparability across risk factors, a counterfactual distribution was defined for each risk factor as the population distribution of exposure that would lead to the lowest levels of disease burden. Thus, for example, in the case of tobacco, this theoretical minimum risk (counterfactual) exposure distribution would be 100% of the population being life-long nonsmokers; for overweight and obesity it would be a narrow distribution of BMI centered around an optimal level (e.g., 21 [with a standard deviation of 1] kg/m^2^), and so on. The theoretical minimum risk exposure distributions for the risk factors quantified in the WHO *Comparative Risk Assessment* study (the risk factor arm of the GBD 2000 study) were developed by expert groups for each risk factor, together with systematic reviews and analyses of extant sources on risk factor exposure and hazard, using an iterative process that increased comparability across risk factors [32],[41]. Results of the *Comparative Risk Assessment* study for the year 2000 are summarized in Table 4.

**Table 4:** The 20 Leading Risk Factor for Deaths and Burden of Disease, World, 2000

Attributable Mortality				Attributable Burden of Disease			
Rank	Risk Factor	Deaths (million)	Percent of Total Deaths	Rank	Risk Factor	DALYs (Millions)	Percent of Total DALYs
1	High blood pressure	7.1	12.8	1	Childhood and maternal underweight	137.4	9.4
2	Smoking and oral tobacco use	4.9	8.8	2	Unsafe sex	91.9	6.3
3	High cholesterol	4.4	7.9	3	High blood pressure	64.3	4.4
4	Childhood and maternal underweight	3.7	6.7	4	Smoking and oral tobacco use	59.1	4.1
5	Unsafe sex	2.9	5.2	5	Alcohol use	58.3	4.0
6	Low fruit and vegetable intake	2.7	4.9	6	Unsafe water, sanitation, and hygiene	54.2	3.7
7	Overweight and obesity (high BMI)	2.6	4.6	7	High cholesterol	40.4	2.8
8	Physical inactivity	1.9	3.4	8	Indoor smoke from household use of solid fuels	38.5	2.6
9	Alcohol use	1.8	3.2	9	Iron deficiency	35.1	2.4
10	Unsafe water, sanitation, and hygiene	1.7	3.1	10	Overweight and obesity (high BMI)	33.4	2.3
11	Indoor smoke from household use of solid fuels	1.6	2.9	11	Zinc deficiency	28.0	1.9
12	Iron deficiency	0.8	1.5	12	Low fruit and vegetable intake	26.7	1.8
13	Urban air pollution	0.8	1.4	13	Vitamin A deficiency	26.6	1.8
14	Zinc deficiency	0.8	1.4	14	Selected occupational risks^a^	21.9	1.5
15	Vitamin A deficiency	0.8	1.4	15	Physical inactivity	19.1	1.3
16	Selected occupational risks^a^	0.8	1.4	16	Lead exposure	12.9	0.9
17	Contaminated injections in health care settings	0.5	0.9	17	Illicit drugs use	11.5	0.8
18	Lead exposure	0.2	0.4	18	Contaminated injections in health care settings	10.5	0.7
19	Illicit drugs use	0.2	0.4	19	Non-use and use of ineffective methods of contraception	8.8	0.6
20	Global climate change	0.2	0.3	20	Child sexual abuse	8.2	0.6

a Includes occupational risk factors for injuries, occupational carcinogens and airborne particulates, ergonomic stressors and occupational noise.

Source: World Health Organization, Comparative Risk Assessment Project [32]

**Figure 4:**
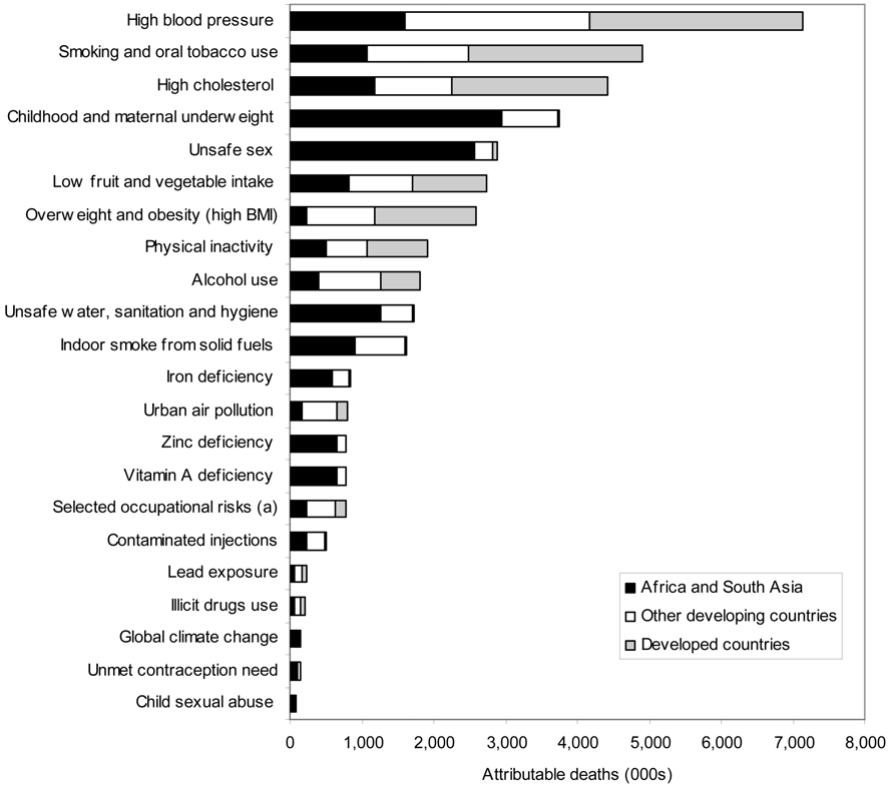
Attributable mortality, by selected major risk factors and region, 2000.

**Figure 5:**
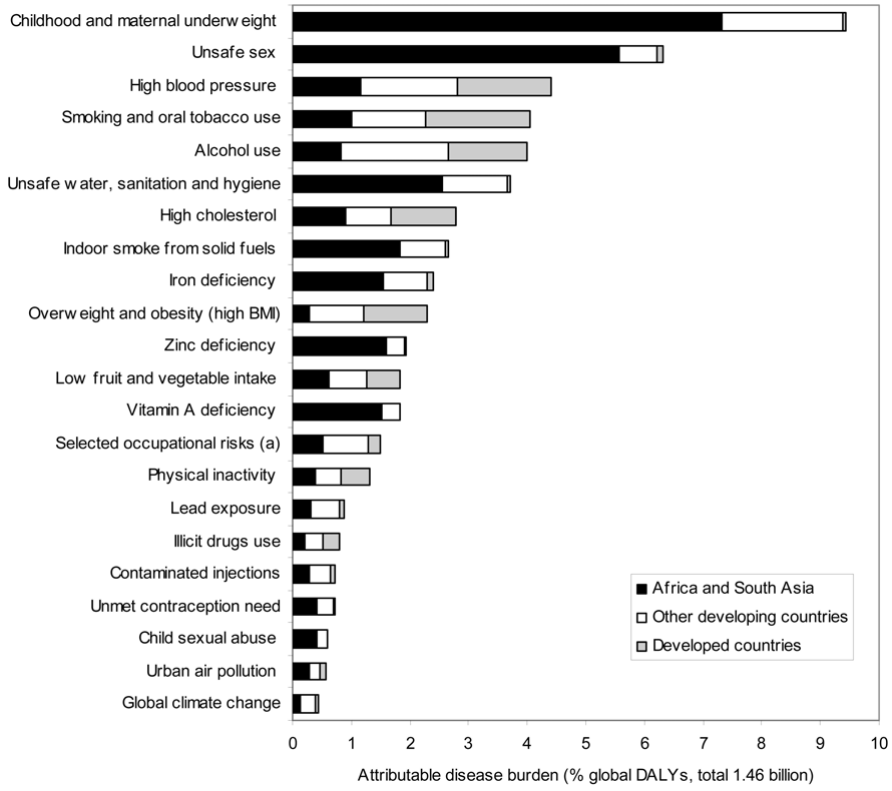
The burden of disease, by selected major risk factors and region, 2000.

The comparative risk assessment for 26 global risk factors, carried out as part of the GBD 2000 study, suggests that risk factors for communicable, maternal, perinatal, and nutritional conditions (e.g., unsafe sex, child and maternal undernutrition, indoor air pollution from household use of solid fuels, and poor water, sanitation, and hygiene)—whose burden is primarily concentrated in the low-income regions of sub-Saharan Africa and South Asia—and risk factors for noncommunicable diseases (e.g., smoking, alcohol, high blood pressure and cholesterol, and overweight and obesity) are leading causes of global disease burden, and that the latter are globally widespread (see Figures 4 and 5).

In developed countries, smoking (12.2%), high blood pressure (10.9%), overweight and obesity (7.4%), alcohol use (9.2%), and high cholesterol (7.6%) were the leading causes of loss of healthy life, contributing mainly to noncommunicable diseases and injuries. In developing countries, leading causes of burden of disease included both risk factors affecting the poor and associated with communicable, maternal, perinatal, and nutritional conditions (e.g., childhood underweight [11.0% of disease burden in these regions], unsafe water, sanitation, and hygiene [4.3%], indoor smoke from household use of solid fuels [3.1%], and unsafe sex [7.3%]), as well as risk factors for noncommunicable diseases (e.g., high blood pressure [3.3%], smoking [2.7%], and alcohol use [3.1%]). Undernutrition was the leading global cause of health loss in 2000, as it was in 1990 (the 2000 results disaggregate undernutrition into underweight and micronutrient deficiencies). Despite substantially improved comparability in GBD 2000, the quantification of risk factor burden needs to expand to include a larger number of risk factors for tropical diseases, injuries, and mental health.

## The GBD 2005: Priorities for a New and Comprehensive Assessment

The Bill & Melinda Gates Foundation has provided funding for a new GBD 2005 study, to be carried out over three years, commencing in 2007. The study will be led by the new Institute for Health Metrics and Evaluation at the University of Washington [42], with key collaborating institutions including Harvard University, the World Health Organization, Johns Hopkins University, and the University of Queensland. This study will also draw on the world's cumulative descriptive epidemiology expertise through a network of around 40 expert working groups. As well as developing new and improved methods to make full use of the increasing amount of health data, particularly from developing countries, the GBD 2005 study will include a comprehensive and consistent revision of disability weights, and assess trends from 1990 to 2005, with projections to 2010. The study will be completed in 2010 and also forms part of the Institute's broader research portfolio on the determinants and outcomes of health system performance assessment.

Disability weights are the crucial link by which conditions that largely cause illness or loss of functional health can be compared with conditions that cause mortality. These weights can have a dramatic effect upon the final estimates, particularly for high-prevalence mild conditions (e.g., hearing loss, visual impairment, anemia, and cognitive impairment, sequelae for a range of infectious and parasitic diseases). The 1990 disability weights were typically estimated for disease sequelae averaged across the distribution of outcomes, in some cases separately for treated and untreated cases, and used groups of health experts rather than general population samples. Some researchers have argued that disability weights for specific diseases or sequelae have been undervalued [43],[44], although in many cases their arguments relate to more severe cases rather than the average of all cases included in the GBD case definitions. The weights have also been criticized by groups interested in particular diseases who have argued that some disabling sequelae have been ignored or undervalued. While some of these concerns are valid, and need to be addressed in the GBD 2005 revisions, it is not always easy to obtain representative population samples of health states associated with given sequelae, particularly those with relatively low prevalence. To prepare consistent and unbiased estimates of YLDs by cause, it was important to ensure that the disability weight and the population incidence/prevalence data relate to the same case definitions.

A particular difficulty is how to measure and characterize the average health states associated with sequelae. This is partly an issue of the lack of information on the population-level distribution of outcomes and the severity of health states. For pragmatic reasons related to the limitations of available data, the original GBD study asked participants to make a composite judgment about the severity distribution of the condition and the preference for time spent in each severity level. The Dutch disability weights project [21] has gone further in assessing disability weights for a range of severity levels of outcomes for a particular sequela (such as mild, moderate, and severe dementia), thus allowing the overall final disability weight for a sequela to take account of regional variations in the severity distribution of outcomes.

The GBD 2005 project will build on methodological advancements [21],[45],[46] and take advantage of new data collection since 1996, which provides extensive cross-cultural data on disability weights. The WHO Multi-Country Household Survey Study in 2001 collected health state valuation data on over 500,000 health states from respondents in 71 countries. This has been used to construct a health state valuation function [46]. The World Health Survey has also included a health state valuation module, and analysis of resulting data is under way [47]. In the next iteration of the burden of disease analyses, it should be feasible to take health state valuations based on such survey data, together with descriptions of outcomes associated with disease sequelae, to produce updated disability weights that take into account not only the available information on health state distributions for disease sequelae, but also the health state preferences of people from all regions of the world.

Although this empirical work provides a much stronger basis for measuring population disability or health state weights, several important research issues remain. Methods for eliciting weights, such as the time trade-off or standard gamble, often capture values other than the level of health associated with a state: the time trade-off method is affected by time preference, the standard gamble method is affected by risk aversion, and choice-based methods may elicit assessments of well-being rather than health [48]. Another particular problem is the measurement of disability weights for conditions of low severity but high prevalence such as anemia and hearing loss, where the current disability weights are small but quite uncertain and multiplied by large prevalences. A disability weight of 0.01 will give half the burden that a disability weight of 0.02 will yield, but no existing valuation measurement method has precision at such low levels of loss of health.

## Discussion and Conclusions

The development and widespread application of a single summary measure of population health (DALYs) has greatly facilitated scientific and political assessments of the comparative importance of various diseases, injuries, and risk factors, particularly for priority setting in the health sector. Comparative rankings of DALYs have led to strategic decisions by some agencies, such as WHO, to invest greater effort in program developments to address priority health concerns such as tobacco control and injury prevention. The subsequent GBD 2000 study, and a plethora of country applications, have led to substantial improvements in both methods and data availability, as well as in the comparability of results. Such global comparative assessments have identified dramatic changes in global health conditions, including impressive reductions in child and adult mortality in many middle-income developing countries and some low-income countries, the explosion of the HIV/AIDS epidemic during the 1990s in sub-Saharan Africa, and the dramatic worsening of adult health and mortality risks in the former Soviet countries in the 1990s.

The comparable analyses of the GBD/Comparative Risk Assessment 2000 frameworks have confirmed the advanced epidemiological transition in most regions for both diseases and their risk factors, with the possible exception of South Asia and Africa. To the unfinished agendas of the neglected tropical diseases, malaria, tuberculosis, HIV/AIDS, and child and maternal mortality have been added new agendas of noncommunicable disease prevention and control, injury prevention and control, and new health threats associated with globalization and trade, particularly tobacco.

The burden of disease methodology and the DALY measure have stimulated considerable debate, particularly in the international and national health policy arenas, among the health economics and epidemiological research communities, and among disability interest groups [13]. Criticisms of the GBD approach fall into three main groups. First, there are concerns about the desirability and implications of extrapolation of population health estimates where data are limited, uncertain, or missing [14]. Second, there has been a lively debate in the literature about the way that the DALY summarizes fatal and nonfatal health outcomes [11],[12]. Third, some well known health economists have argued that burden of disease analysis is irrelevant or potentially misleading for setting health priorities [11],[49].

Murray and colleagues have argued that health planning, including that based on uncertain assessments of evidence that synthesizes the available data and information while ensuring consistency and adjustment for known biases, will almost always be more informed than planning based on ideology, special interests, or crude statistics, which are often biased and inconsistent [50]. Murray has recently clarified the roles of crude, corrected, and predicted health statistics [51]. While we strongly advocate that corrected and predicted health statistics should be used to produce a comprehensive and unbiased picture of the global burden of disease for health policy and planning, we suggest that evaluation and monitoring of health systems and interventions, on the other hand, should be based on corrected, but not predicted, statistics.

The DALY has received a great deal of criticism from disability advocates and some health analysts, who have interpreted the inclusion of disability in the DALY as implying that people with disability are less valued than people in full health [52]. The WHO definition of health is grounded in a multidimensional notion of health functioning, and the conceptual basis for the DALY has moved from an original somewhat ill-defined focus on the “social value of health” [23] to a focus on quantifying the loss of health per se rather than the quality of life or well-being associated with that loss of health [53]. Loss of health is conceptualized in terms of domains of health functioning, which include body functions such as fertility, respiration, vision, or pain, as well as more complex functions such as mobility, affect, or cognition. While definitions and concepts of disability vary widely across societies, the WHO International Classification of Functioning, Disability and Health [54] defines “disability” as an umbrella term for impairments, activity limitations, and participation restrictions that are health-related. According to this definition, anyone who is disabled has a reduction in functional health and thus does not have “full health” in terms of the functional health concept that the DALY quantifies.

Some disability advocates have explicitly rejected this viewpoint [52], apparently equating health with the absence of active disease or pathology (in our view a narrowly medical conceptualization of health). This probably reflects concerns that if people with disabilities are seen as having reduced health, this may increase discrimination in the allocation of resources. We would argue that metrics for quantifying population health loss should incorporate losses in health functioning: a population with high levels of onchocerciasis-caused blindness has less health than one with low levels, even if the current prevalence of onchocerciasis infection is zero in both. The disability weights used in the GBD have also been incorrectly criticized as implying that every person with a particular health condition experiences the same health state [52]. Of course, the average disability weights used in the GBD are intended to represent the average health loss at population level only, not individual level.

As used in the DALY, the term “disability” is essentially a synonym for states of less than full health, conceptualized in terms of severity-adjusted functional health loss. The term disability was chosen to stress a vision of health that goes beyond the absence of disease to include decreases in functioning resulting from disease. While use of a term such as health-adjusted life years would perhaps be more accurate, DALYs are widely used and a name change now would lead to more confusion than clarity. However, the DALY quantifies loss of health, and the disability weights are thus intended to reflect social preferences for health states, not broader valuations of “quality of life,” “wellbeing,” or “utility” [53]. A high disability weight for a health state then implies that people place a high social value on preventing such health states and says nothing whatsoever about the wellbeing, quality of life, or value of the people experiencing such health states.

Some health economists have expressed concern that burden of disease analysis might result in priority setting solely on the basis of the magnitude of disease burden, arguing that burden of disease studies are irrelevant for priority setting and that all one needs to know is the marginal cost-effectiveness of potential interventions [49]. Although this view has little credibility among policy makers, who are generally very interested in understanding the patterns and causes of health loss in populations and their changes over time, it is in fact a misrepresentation of the purpose of burden of disease analysis. The original GBD study, the later round of GBD work at WHO, and the use of GBD results in the Disease Control Priorities Project have all been accompanied by substantial efforts in cost-effectiveness analysis, and an explicit recognition that health priority setting requires not only information on the size and causes of health problems, but on the cost-effectiveness of interventions, and on other information relating to equity and social values [1],[55],[33]. Further, using a quantification of the GBD based on DALYs and on the analysis of the health gains from various intervention investments does not in any way imply that the user ascribes to the view that health resources should be strictly allocated to maximize health. In fact, the WHO framework for health system performance assessment explicitly included substantial emphasis on reducing health, financial, and other inequalities as an objective of health systems [56].

Widespread use of published summary measures provides clear evidence that there is a demand for the simplification of epidemiological complexity that summary measures provide. Of course, the provision of summary measures does not preclude the full dissemination of the underlying internally consistent incidence, prevalence, and mortality estimates. In particular, there is considerable demand for a revised GBD study that reliably measures changes in global health and disease patterns over the past 15 years or so. More money is being spent on global health than ever before—both by governments, private foundations, and nongovernmental organizations. Advocacy groups have appreciated the value of good comparative statistics to galvanize public support and policies, as reflected by the increasing interest in the neglected tropical diseases. Additionally, donors and others in the global health community are increasingly demanding a greater understanding of trends in health in order to better allocate their resources and make real progress in improving health. Critical policy questions depend upon understanding trends. Is malaria mortality in children in Africa increasing in the context of rising chloroquine drug resistance or not? Has there been a resurgence of onchocerciasis in parts of Africa? Has there been a decline in HIV mortality in populations with significant antiretroviral treatment coverage? How much progress is being made in the elimination of diseases such as lymphatic filariasis and human African trypanosomiasis? Which populations are missing out on access to effective treatments for helminthic infections? Does research funding and priority setting neglect some areas of high disease burden? These are important policy questions that require new, critical analyses of the type provided by the GBD framework. The new GBD study will also revise 1990 estimates using consistent data and methods to assess trends in the global burden of diseases and injuries from 1990 to 2005.

A particular challenge for the new GBD study will be the comparative lack of information for tropical and neglected diseases. Data availability may have worsened for some diseases. The GBD 2000 malaria estimates and estimates for some of the key causes of child death were forced to draw on studies of incidence and case fatality from the 1980s and 1990s. This appears to reflect a decline in interest by either investigators or journals in descriptive epidemiology studies that may not be sufficiently “novel” for funder and journal audiences. An additional challenge for comparative risk assessment in relation to the tropical diseases is that “risk factors” for tropical diseases often have highly heterogeneous effects across populations: for example, the risk from not using bed-nets is highly dependent on housing and on local ecological and meteorological factors; similarly the effects of each environmental or socioeconomic risk factor on the prevalence of tropical diseases such as schistosomiasis and hookworm depends on coexistence of other risks and on geographical factors [57]. Yet comparative measurement of the effects of risk factors for disease and injury has significant policy potential and thus should be further explored for this group of diseases. Therefore the analytical and empirical work on comparative risk assessment should be expanded to include risk factors for tropical diseases. Key research priorities for improving our understanding of the burden of neglected tropical diseases are listed in Box 1.

**Box 1. Future Research Directions**

Key issues for future research on the burden of neglected tropical diseases include:

- Assessing the need to explicitly address additional diseases not currently included in the GBD. The current draft cause list for the GBD 2005 also includes cysticercosis, echinococcosis, dracunculiasis, yellow fever, rabies, and leptospirosis.
- Review of the disease sequelae quantified for each disease to ensure that all important disabling outcomes are captured, and also that the natural history of the disease is appropriately modeled.
- Development of improved disease models for the estimation of incidence and duration. For several important diseases, the current GBD study does not attempt to estimate incidence, and effectively assumes that incidence equals prevalence for the calculation of YLDs.
- Comprehensive revision of disability weights for disabling sequelae incorporating population-level information on the distribution of health states.
- Addressing the issue of so-called subtle morbidity, i.e., small decrements in functioning (e.g., fatigue) associated with chronic infection.
- Development of methods for the assessment of disability weights for highly prevalent impairments or sequelae of low average severity (e.g., anemia, cognitive deficits).
- Development of methods to ensure that disease-specific estimates of impairments common to a number of disease and injury causes, such as anemia or cognitive deficits, collectively match population-level total prevalences for such impairments.
- Addressing the difficult issues of assessing the incidence and prevalence of highly focal diseases. Studies tend to focus on areas with disease—how representative are these studies of the whole population at-risk, what populations are at risk, how to extrapolate to national, regional, and global estimates of incidence and prevalence?
- Estimating attributable deaths due to NTDs for long-term outcomes such as cancers, cirrhosis of the liver, and renal failure.
- Estimating cause-specific mortality for diseases with relatively low case fatality rates in regions without useable death registration data. Innovative new approaches to the use and validation of verbal autopsy instruments may be helpful.
- Identifying key risk factors for NTD incidence and mortality, quantifying exposure distributions for individual and multiple risk factors, and quantifying their hazardous effects, especially when the hazardous effects may depend on the presence of other risks.

As international programs and policies to improve health worldwide become more widespread, so too will the need for more comprehensive, credible, and critical assessments to periodically monitor population health and the success, or otherwise, of these policies and programs. Repeated one-off assessments of the global burden of disease do not provide comparability over time due to improvements in data and methods. There is a need to move beyond these, towards truly consistent and comparable monitoring of the world population's health over time. We thus welcome the forthcoming series of reviews on the burden of neglected tropical diseases to be published in this journal, as an important contribution to this task.

## Supporting Information

Table S1 Country groupings used in this paper
(0.06 MB DOC)
