# Supplementary material for: Measuring the Burden of Neglected Tropical Diseases: The Global Burden of Disease Framework
Source: PLoS Negl Trop Dis. 2007 Nov 7;1(2):e114. doi: 10.1371/journal.pntd.0000114 (PMC2100367; doi:10.1371/journal.pntd.0000114)
Supplement: Table S1 — Country groupings used in this paper (0.06 MB DOC) [file pntd.0000114.s001.doc]

Supplementary Table S1: Country regional groupings used

| Country grouping | WHO subregions (a) | WHO Member States |
| --- | --- | --- |
| Low mortality countries (b) | AMRO A, EURO A, WPRO A | Canada, United States Of America, Cuba, Andorra, Austria, Belgium, Croatia, Czech Republic, Cyprus, Denmark, Finland, France, Germany, Greece, Iceland, Ireland, Israel, Italy, Luxembourg, Malta, Monaco, Netherlands, Norway, Portugal, San Marino, Slovenia, Spain, Sweden, Switzerland, United Kingdom, Australia, Japan, Brunei Darussalam, New Zealand, Singapore |
| Europe and Central Asia (b) | EURO B,  EURO C | Albania, Armenia, Azerbaijan, Bosnia And Herzegovina, Bulgaria, Georgia, Kyrgyzstan, Poland, Romania, Slovakia, Tajikistan, The Former Yugoslav Republic Of Macedonia, Turkey, Turkmenistan, Uzbekistan, Yugoslavia, Belarus, Estonia, Hungary, Kazakhstan, Latvia, Lithuania,  Republic of Moldova, Russian Federation, Ukraine |
| Latin America and Caribbean | AMRO B, AMRO D | Antigua and Barbuda, Argentina, Bahamas, Barbados, Belize, Bolivia, Brazil, Chile, Colombia, Costa Rica, Dominica, Dominican Republic, Ecuador, El Salvador, Grenada, Guatemala, Guyana, Haiti, Honduras, Jamaica, Mexico, Nicaragua, Panama, Paraguay, Peru, Saint Kitts and Nevis, Saint Lucia, Saint Vincent and The Grenadines, Suriname, Trinidad and Tobago, Uruguay, Venezuela |
| Eastern Mediterranean Region | EMRO B, EMRO D | Afghanistan, Bahrain, Djibouti, Egypt, Iran (Islamic Republic Of), Iraq, Jordan, Kuwait, Lebanon, Libyan Arab Jamahiriya, Morocco, Oman, Pakistan, Qatar, Saudi Arabia, Somalia, Sudan, Syrian Arab Republic, Tunisia, United Arab Emirates, Yemen |
| East Asia, SE Asia and Pacific | SEARO B, WPRO B | Cambodia, China, Indonesia, Lao People's Democratic Republic, Malaysia, Mongolia, Philippines, Republic Of Korea, Sri Lanka, Thailand, Viet Nam |
|  |  | Cook Islands, Fiji, Kiribati, Marshall Islands, Micronesia (Federated States Of), Nauru, Niue, Palau, Papua New Guinea, Samoa, Solomon Islands, Tonga, Tuvalu, Vanuatu |
| South Asia | SEARO D | Bangladesh, Bhutan, Democratic People's Republic Of Korea, India, Maldives, Myanmar, Nepal |
| AFRO | AFRO D,  AFRO E | Algeria, Angola, Benin, Burkina Faso, Cameroon, Cape Verde, Chad, Comoros, Equatorial Guinea, Gabon, Gambia, Ghana, Guinea, Guinea-Bissau, Liberia, Madagascar, Mali, Mauritania, Mauritius, Niger, Nigeria, Sao Tome and Principe, Senegal, Seychelles, Sierra Leone, Togo, Botswana, Burundi, Central African Republic, Congo, Côte d'Ivoire, Democratic Republic Of The Congo, Eritrea, Ethiopia, Kenya, Lesotho, Malawi, Mozambique, Namibia, Rwanda, South Africa, Swaziland, Uganda, United Republic of Tanzania, Zambia, Zimbabwe |

Note (a) The six WHO regions have been divided into 14 subregions, based on five mortality strata A-E, for GBD reporting in WHO World Health Reports (refer to World Health Report 2004, page 156).

b) Low mortality countries and Europe and Central Asia are referred to collectively as "Developed countries" and all other countries as "developing countries":
